# New STAT3-FOXL2 pathway and its function in cancer cells

**DOI:** 10.1186/s12860-019-0206-3

**Published:** 2019-06-20

**Authors:** Yangyang Han, Jun Wu, Weiwei Yang, Di Wang, Tianliang Zhang, Min Cheng

**Affiliations:** 1 School of Bioscience and Technology, Weifang Medical University, Weifang, Shandong 261053 People’s Republic of China; 2 Plastic Surgery Institute of Weifang Medical University, Weifang, Shandong 261053 People’s Republic of China; 3 Experimental Center for Medical Research, Weifang Medical University, Weifang, Shandong 261053 People’s Republic of China; 4 Department of Physiology, Weifang Medical University, Weifang, Shandong 261053 People’s Republic of China

**Keywords:** Cell apoptosis, Signal transduction, STAT3-FOXL2 pathway, Transcriptional regulation

## Abstract

**Background:**

The forkhead transcription factor (FOXL2) plays a crucial role in blepharophimosis-ptosis-epicanthus inversus syndrome (BPES), sex determination, ovary growth and development, and cell cycle regulation. Emerging investigations have focused on the downstream targets of FOXL2, while little is known about its upstream regulation.

**Results:**

In this study, we show that FOXL2 could be regulated by STAT3 in cancer cells and that STAT3 binds to FOXL2 at the 5′- GCCTGATGTTTGTCTTCCCAGTCTGTGGCAA-3′ site using EMSA and ChIP. We further found that knockdown of STAT3 or FOXL2 could significantly induce cancer cell apoptosis, indicating the importance of these two genes in cancer cell growth and apoptosis. Our data also indicated that the increased apoptotic cell rate may be caused by changes in apoptosis-related genes, such as *TNF*, *TRAIL* and *GnRHR*.

**Conclusion:**

This study presents a new upstream regulator of FOXL2 and demonstrats that this new STAT3-FOXL2 pathway has an important function in HeLaHeLa cell apoptosis, providing new insights regarding the targeting of FOXL2 for cancer prevention and treatment.

**Electronic supplementary material:**

The online version of this article (10.1186/s12860-019-0206-3) contains supplementary material, which is available to authorized users.

## Background

Forkhead box L2 (FOXL2) is a gene encoding a forkhead transcription factor that belongs to the forkhead/winged-helix transcription factor superfamily. FOXL2 is a single-exon gene that encodes a 376 amino acids protein in humans that contains a 110-amino-acid DNA-binding forkhead domain (FHD) and a polyalanine (poly-Ala) tract of 14 residues of unknown function [1], and this gene is preferentially expressed in the ovary, the eyelids and the pituitary gland [2].

Many researchers have focused on the downstream targets of FOXL2 as a major transcriptional factor. To data, the potential direct and indirect FOXL2 transcriptional targets include a sex determination gene (*SOX9*) [3], ovarian development and granulosa cell differentiation related genes (e.g., *FST* and *CDKN1B*) [4, 5], estrogen production and steroidogenesis-related genes (e.g., *CYP19A1*, *CYP17A1*, and *StAR*) [6–8], ovulation-related genes (e.g., *SERPINE2*, *HAS2* and *PTGER2*) [9], signal transduction related genes (e.g., *SMAD3*, *BMPR1A* and *DKK3*) [5], and apoptosis related genes (e.g., *TNF-R1*, *FAS*, *TRAIL-R*, *BCL2A1* and *FOS*) [10, 11]. In addition, the potential transcriptional targets of FOXL2 include cell cycle related genes and stress response related genes in humans [12].

However, the upstream regulation of FOXL2 has not been thoroughly elucidated to date. The few papers describing this regulation indicated that miR-133a and miR-133b could bind to the 3’UTR region of FOXL2 mRNA and reduce its mRNA and protein expression levels [13, 14]. In 2017, Yu et al. demonstrated that microRNA-937 could inhibit cell proliferation and metastasis in gastric cancer cells by downregulating FOXL2 [15]. However, research papers investigating the upstream regulation of *FOXL2* are still limited and restrict the further study of the biological functions of the gene.

As FOXL2 is a major transcriptional factor involved in many important biological processes, especially in ovary differentiation, both its up- and downstream regulation are similarly important. In our previous paper (Han et al., 2017), our results indicated that there was a predicted binding site of STAT3 (signal transducer and activator of transcription 3) in the promoter region (1931 bp) of FOXL2 using bioinformatics, and studies have indicated that both STAT3 and FOXL2 have similar expression patterns in tissues. For example, a transcriptome analysis found that STAT3 signaling was extensively enriched in granulosa cell compartment of human primordial and primary follicle [16], apart from the already known mechanistic targets, such as FOXL2. In addition, the activated STAT3 signal was also detected in corneal epithelium, stroma [17] and pituitary [18], which were formally demonstrated that were the main expression tissues of FOXL2.

However, although it is possible that FOXL2 was regulated by STAT3 because of the similar tissue expression and our previously bioinformatics results, the precise sequences that STAT3 binds in the promoter region of FOXL2 are still unknown. In addition, considering that STAT3 is persistently activated in many human cancer tissues and cell lines [19], if FOXL2 is clearly regulated by STAT3, the question remains of whether the new STAT3-FOXL2 signaling pathway functions cancer progression.

In this paper, we mainly focus on upregulation of FOXL2, the chromatin immunoprecipitation (ChIP) and electrophoretic mobility shift assay (EMSA) results demonstrated that there are accurate STAT3 binding sequences (5′-GCCTGATGTT**TGTCTTCCCAGTCTGT**GGCAA-3′) in the promoter region of *FOXL2* for the first time. Further results indicated that the STAT3-FOXL2 pathway played a major role in cervical cancer cell growth and apoptosis using RNA interference, and it may be caused by the changed expression level of the related apoptotic genes.

## Results

### Accurate binding sequence of STAT3 in the promoter region of FOXL2

**Fig. 1:**
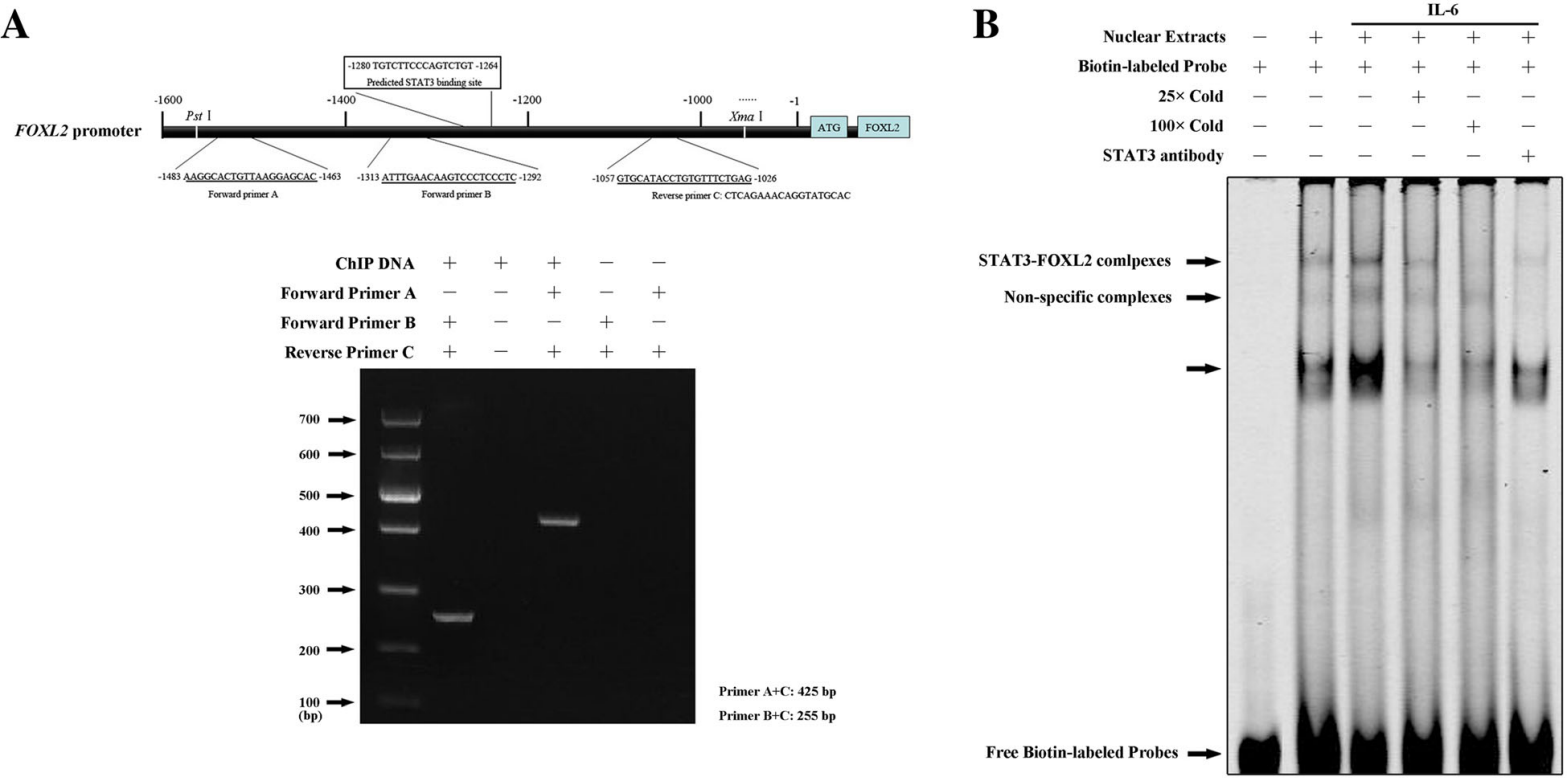
Results of ChIP and EMSA demonstrate that *FOXL2* is regulated by STAT3. **a** ChIP demonstrates that anti-STAT3 antibodies immunoprecipitate *FOXL2*. The *FOXL2* gene is detected in nuclear protein (stimulating with human IL-6) immunoprecipitated with anti-STAT3 antibody using PCR (down), and the detected primers used in the PCR are designed as shown (above), demonstrating that STAT3 binds to the *FOXL2* promoter which contains the predicted sites. **b** EMSA results using biotin-labeled and unlabeled probes that contain STAT3 predicted binding sites, show that nuclear proteins bind to the biotinylated DNA fragments and that the addition of the corresponding cold DNA fragment (unlabeled probes) or anti-STAT3 antibody attenuates this binding

In our previous paper, we demonstrated that the luciferase activity fused to the promoter of *FOXL2* was significantly downregulated when HeLa cells were treated with a STAT3 inhibitor, suggesting that STAT3 activated the *FOXL2* gene. However, the precise binding site was not determined. To further validate the interaction between STAT3 and FOXL2, we performed ChIP and EMSA. First, we used ChIP to determine whether STAT3 directly binds the predicted STAT3 binding element in the *FOXL2* promoter. We obtained nuclear extracts of HeLa cells (IL-6-stimulated) and used ChIP and PCR to assess the binding of STAT3 to the predicted STAT3 binding site (5′-**TGTCTTCCCAGTCTGT**-3′). As shown in Fig. 1a, we found that primers A + C and primers B + C, corresponding to the putative STAT3-binding site depicted in Fig. 1a (above), could amplify PCR products with DNA fragments that coimmunoprecipitated with anti-STAT3 antibodies. The same primers A + C and primers B + C without DNA fragments amplified nonproducts. These results confirm that the STAT3 binding site is between primers B and C (255 bp) in the FOXL2 promoter, which is consistent with the predicted STAT3 binding sites obtained using bioinformatics.

Then, to further validate these findings, we performed an electromobility shift assay (EMSA). As shown in Fig. 1b, HeLa nuclear protein bound the biotinylated probe in the *FOXL2* promoter fragment (5′ Biotin-GCCTGATGTT**TGTCTTCCCAGTCTGT**GGCAA-3′), and excess cold probes (25× or 100×) attenuated STAT3-FOXL2 complexes, In addition, anti-STAT3 antibodies showed similar attenuated binding complexes with the cold probes. The result in Fig. 1b suggested that the STAT3 binding site was within the 31-bp probe in the promoter of *FOXL2* which contained our previously predicted sequence.

### Knockdown of p-STAT3 and FOXL2 by STAT3 siRNA

**Fig. 2:**
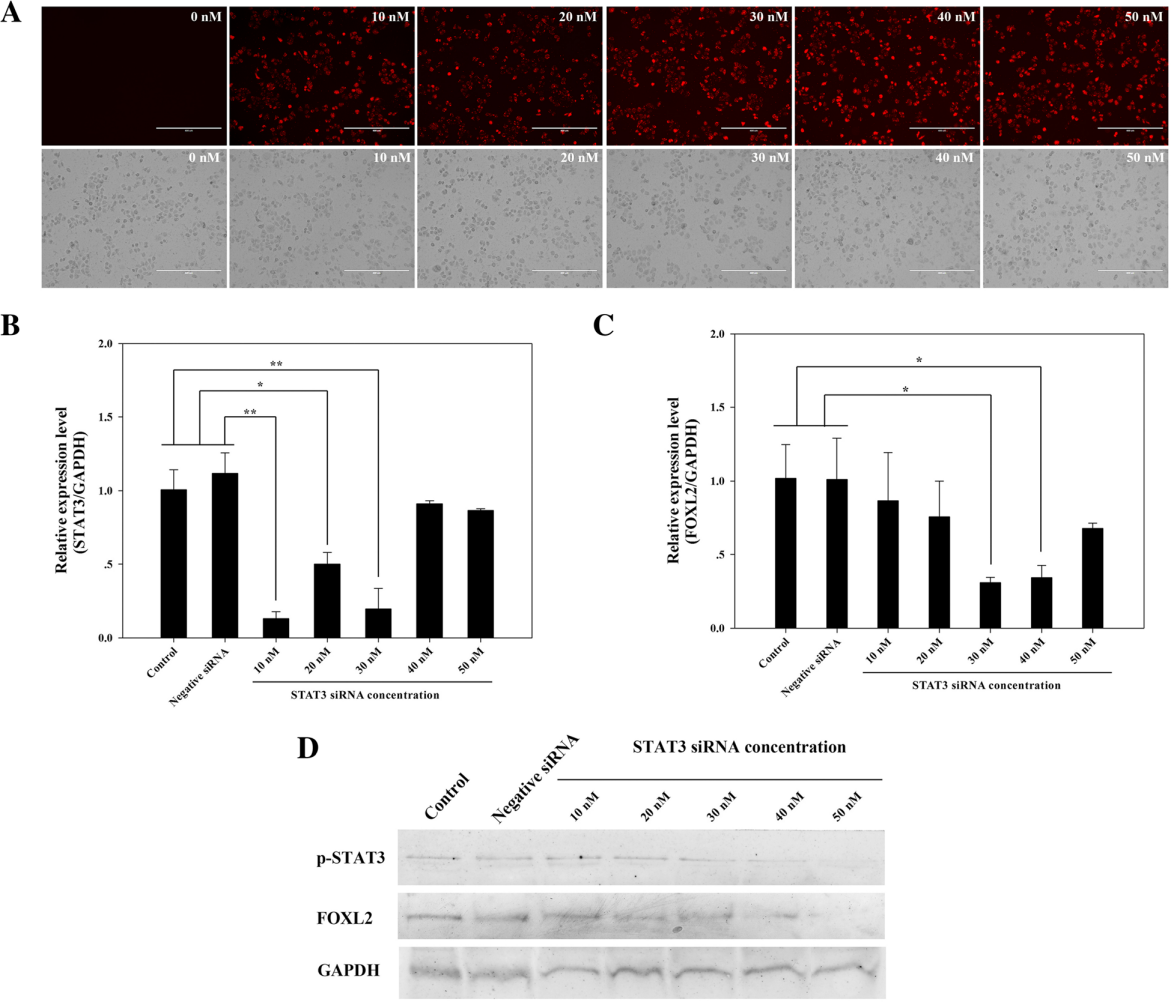
Transfection with STAT3 siRNA inhibits the expression of phosphorylated-STAT3 and FOXL2. **a** Lipofectamine 3000 and the Red Fluorescent Control complexes (doses range from 0 to 50 nM) indicate that HeLa cells have relatively higher transfection efficiencies at 30 nM. **b**, **c** qRT-PCR demonstrated that STAT3 siRNA downregulates the mRNA levels of phosphorylated *STAT3* and *FOXL2* relative to *GAPDH*. The cells with no transfection or transfected with negative siRNA (Thermo Fisher) were both used as controls. Each column represents the mean ± SEM of at least three replicates, and the whole experiment was repeated 3 times, * and ** indicate significant differences at *P* < 0.05 or *P* < 0.01, respectively. **d** Western blotting demonstrates that STAT3 siRNA reduces p-STAT3 and FOXL2 protein levels in a dose dependent manner, whereas the control and negative siRNA show almost no reduction

To find the best transfection efficiency, the BLOCK-IT Alexa Fluro Red Fluorescent Control, with doses ranging from 0 to 50 nM, was used in the pretransfection. The results in Fig. 2a indicated that all concentrations of fluorescent control satisfied the transfected efficiency, and the 30-nM dose was better than 10- and 20-nM doses, and similar with 40- and 50-nM doses. Then, to evaluate the ability of STAT3 siRNA knockdown, according to the manufacturer’s suggestion, HeLa cells were transfected with siRNA doses ranging from 10 to 50 nM. The results in Fig. 2b indicated that the mRNA expression level of STAT3 was downregulated after transfection with STAT3 siRNA in a dose independent manner compared with the control and negative siRNA transfection, and showed a significant decrease in doses ranging from 10 to 30 nM. Meanwhile, the protein level of p-STAT3 was clearly knocked down as the dose of STAT3 siRNA increased (Fig. 2d).

As we expected, both the mRNA (Fig. 2c) and protein (Fig. 2d) expression of FOXL2 showed a similar knockdown of STAT3 after the HeLa cells were transfected with STAT3 siRNA at different doses.

### Knockdown of STAT3 or FOXL2 changes the fate of cells

**Fig. 3:**
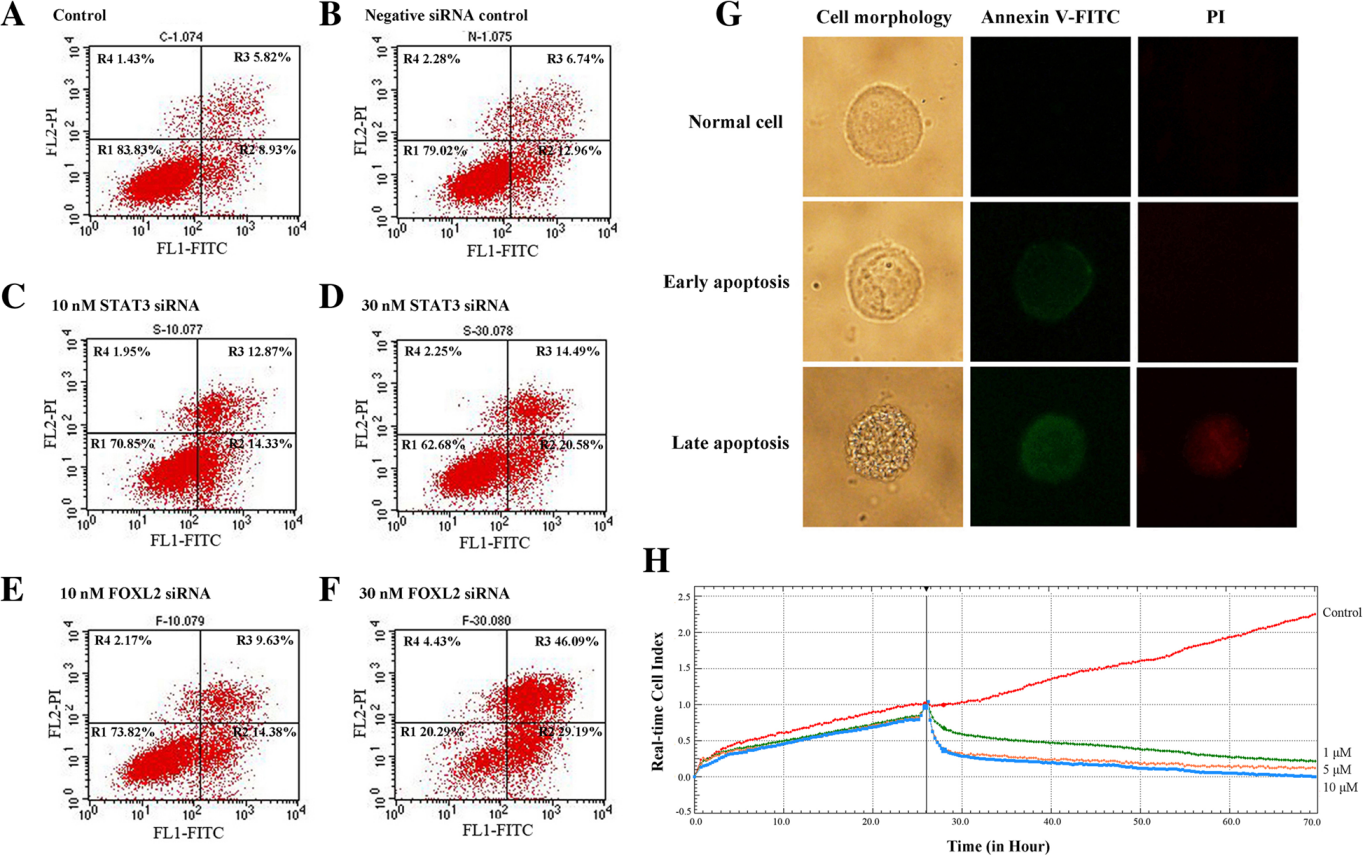
STAT3 and FOXL2 are both involve in HeLa cell apoptosis. The effects of STAT3 and FOXL2 on cancer cell apoptosis were detected using flow cytometry detection. Cells transfected with different concentrations of STAT3 siRNA (**c**, **d**) and FOXL2 siRNA (**e**, **f**) exhibited relatively higher R2 and R3 rates than control (**a**) and negative siRNA transfection (**b**). FL1-FITC: Annexin V-FITC was used to detect early apoptosis. FL2-PI: propidium iodide (PI) to detect late apoptosis. R1: living cells, R2: early apoptosis, R3: late apoptotic cells, R4: dead cells. **g** Cell morphology of Annexin V-FITC and PI staining during apoptosis. **h** Real-time cell viability was detected after treatment with STAT3 inhibitor. Normal HeLa cells were plated onto an electrode-containing plate (e-plate) where they adhered for 24 h, and then were treated with 1, 5 or 10 μM concentrations of various STAT3 inhibitors. Cell viability was tracked using the xCelligence real-time viability system, and each experiment was repeated at least two times

To further study the functions of *STAT3* and *FOXL2* in HeLa cells, cell fate was examined by flow cytometry and RTCA. As shown in Fig. 3a and b, cells under normal conditions (Fig. 3a) or transfected with negative siRNA (Fig. 3b) were both used as controls to compensate for the fluorescence. Compared with controls, both 10 nM and 30 nM STAT3 siRNA transfection induced increased early apoptosis and late apoptosis rates (Fig. 3c and d). Similar to STAT3 siRNA transfection, there were more early and late apoptotic cells after transfection with FOXL2 siRNA, especially with the dose of 30 nM FOXL2 siRNA (Fig. 3f). These data indicated that STAT3 and FOXL2 knockdown induced apoptosis, in HeLa cells.

The cell morphologies in the above treatments were examined to observe apoptosis (Fig. 3g). Normal cells were not stained by Annexin V-FITC, because phosphatidylserine (PS) was only distributed in the cell membrane lipid bilayer. The early apoptotic cells were stained by Annexin V-FITC as green fluorescence because their PS was turned toward the outside of the lipid membrane, thereby binding Annexin V-FITC. The late apoptotic cells were both stained with green fluorescence by Annexin V-FITC and red by PI because the cell membrane was damaged, and the PI could bind to the nucleic acid.

Then, the real-time cell viabilities (cell index) were detected under treatment with different concentrations of STAT3 inhibitor, and cells treated with DMSO were used as a control. In keeping with our prediction, cell indexes showed a marked decline after HeLa cells were exposed to different doses of inhibitor compared with control cells (Fig. 3h). The inhibition of the cell index showed a dose-depended action of the inhibitor (Fig. 3h).

### Changed mRNA expression of the related genes may be the reasons for the changed cell fate

**Fig. 4:**
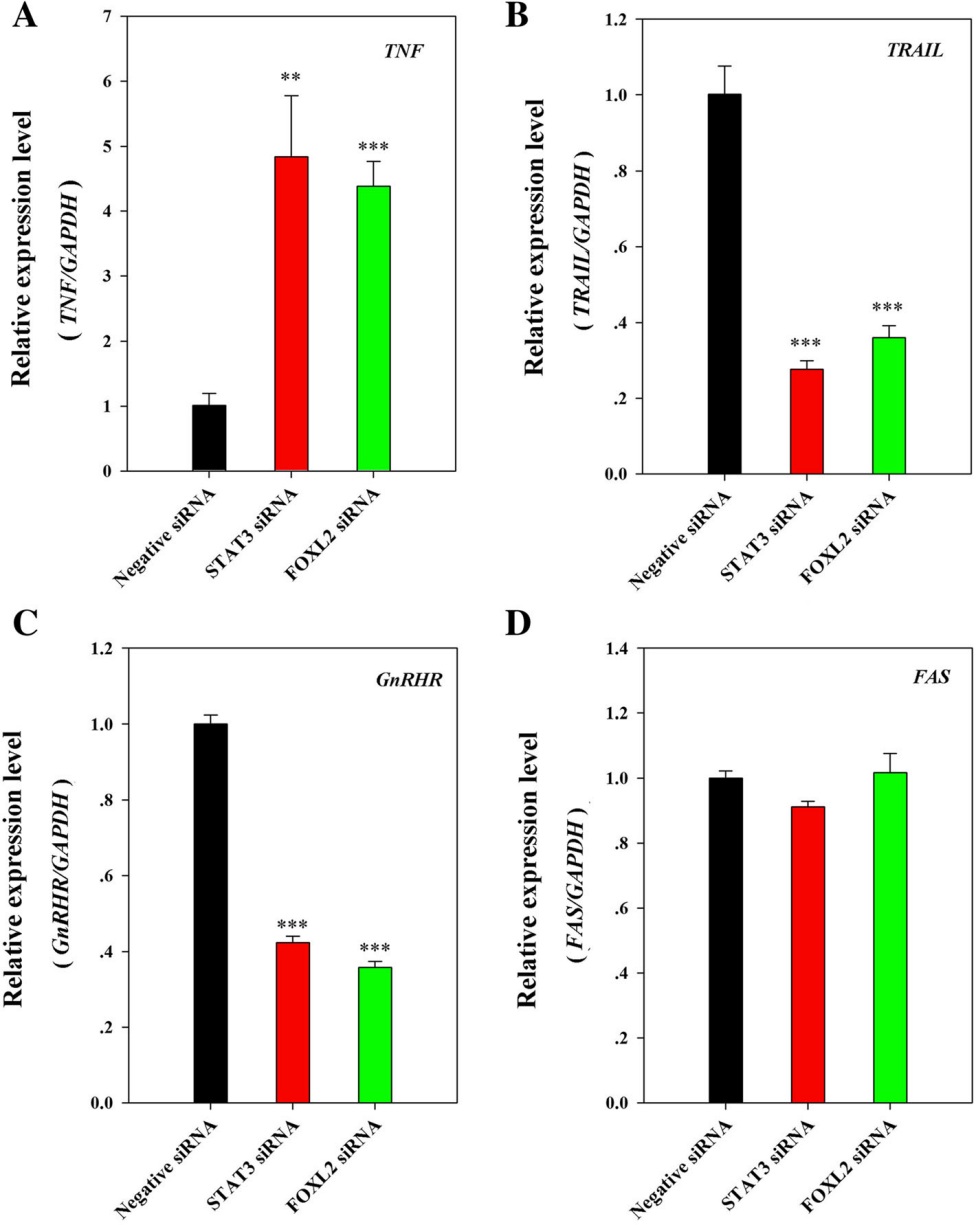
mRNA expression levels of apoptosis-related genes are influenced by transfection with STAT3 or FOXL2 siRNA. The related mRNA levels (**a**, **b**, **c**, **d**) in cells transfected with STAT3 or FOXL2 siRNA (30 nM) are indicated relative to cells transfected with negative siRNA (30 nM), referring to the transcript of *GAPDH* in the same sample. Each column represents the mean ± SEM of three replicates, and the entire experiment was repeated 3 times, ** and *** indicate significant differences at P < 0.01 or *P* < 0.001, respectively

To further investigate the reasons for cell fate changes, qRT-PCR was performed to determine apoptosis-related genes. The same concentrations (30 nM) STAT3 siRNA or FOXL2 siRNA were transfected into HeLa cells, and the negative siRNA which was directly purchased from Thermo Fisher Scientific was used as a negative control. Among these detective apoptosis related genes, the *TNF* expression level was significantly up-regulated after transfection with STAT3 or FOXL2 siRNAs compared to the negative control (Fig. 4a). The mRNA levels of *TRAIL* and *GnRHR* showed a very similar expression pattern and were both downregulated after transfection with STAT3 or FOXL2 siRNAs compared with the negative control (Fig. 4b and c). The expression of *FAS* showed no clear changes between STAT3 or FOXL2 siRNA-transfected samples and the control sample (Fig. 4d). In addition, the results in Fig. 4 indicate that the apoptosis related genes showed very similar expression after transfection with STAT3 siRNA or FOXL2 siRNA, indicating that these genes were regulated not only by FOXL2 but also by STAT3.

## Discussion

As an essential transcription factor, FOXL2 is not only involved in the normal development of the ovary and the eyelid [20], but is also related to steroid metabolism, reactive oxygen species detoxification, estrogen production, stress response and inflammatory processes [21]. In addition, FOXL2 mutations were reported to be associated with polycystic ovary syndrome (PCOS) [22] and blepharophimosis-ptosis-epicanthus inversus syndrome (BPES) [23, 24]. Among those processes, researchers mainly focus on the downstream regulation of *FOXL2* while ignoring the upstream regulation, and there are only a small number of studies regarding this regulation to date. It was determined that microRNA-937, microRNA-133a and microRNA-133b could bind to the 3′-untranslated region (3′-UTR) of *FOXL2* mRNA, and both mRNA and protein expression could be downregulated by these factors [13–15]. In 2017, in a new result, Dong et al. reported that FOXL2 could be regulated by HMGA2, a member of the high motility group (HMG) protein family [25].

We focus on the upstream regulation of FOXL2 because the lack research on this regulation has prevented a full functional-understanding of this major gene from being achieved. Our previous paper implied that there was a potential binding site of STAT3 in the promoter region of *FOXL2* using bioinformatics (Additional file 1: Table S1) and demonstrated the regulation of FOXL2 by STAT3 via luciferase reporter assays [26]. Furthermorem, in this paper, the direct interaction between STAT3 and FOXL2 was demonstrated using EMSA and ChIP assays (Fig. 1), suggesting a new STAT3-FOXL2 pathway for the first time and providing a new insight into the two related genes.

On the one hand, as a new upstream regulator of FOXL2, STAT3 may play a role by regulating FOXL2, especially in the development of ovaries. Lee et al. reported that Obox4-silencing-induced premature STAT3 activation at the germinal vesicle (GV) stage provoked subsequent GV breakdown, while STAT3 activation is sufficient for stimulating the continuation of meiosis in mouse oocytes, indicating the crucial role of STAT3 in oocytes [27]. A similar function of STAT3 was found in granulosa cell death and follicular atresia [28]. Considering the extremely important role of FOXL2 in follicle activation and granulosa cell differentiation, it is possible that FOXL2 is an effector of STAT3 in the whole regulation process.

On the other hand, considering the crucial roles of STAT3 in various cancers, as a new downstream regulator gene of STAT3, FOXL2 may have an important function in cancer. There is considerable direct evidence suggesting the crucial role of STAT3 in various processes, including cancer growth, invasion, and apoptosis [29–32], and it can be activated by various proto-oncogenes and oncogenes [33–37]. As a the new downstream gene of STAT3, our results demonstrated that knockdown of STAT3 or FOXL2 promoted apoptosis and inhibited the growth of cervical cancer cells using RNAi (Fig. 3). Our further research indicated that the altered apoptosis and growth of cancer cells might be caused by the related genes that are downstream and regulated by FOXL2, especially tumor necrosis factor (*TNF*) (Fig. 4). Not only in cervical cancer, Dong et al. reported that the new HMGA2-FOXL2 pathway was involved in the regulation of multiple malignant behaviors in gastric cancer and confirmed that the inhibition of FOXL2 expression impaired cell migratory, invasion potential, and epithelial-to-mesenchymal transition (EMT) [25]. MicroRNA-937-FOXL2 is also involved in cell proliferation and metastasis in gastric cancer [15]. Moreover, reports have indicated that FOXL2 may function in sex cord stromal tumors [38].

Current research indicates that FOXL2 plays a major role in granulosa cell tumors (GCTs), and the 402C > G mutation in *FOXL2* is crucial to the development of adult GCTs [39–42]. However, there is still debate about the function of this gene as a of tumor suppressor. In cancer, the patterns of mutations in well-studied oncogenes and tumor suppressor genes are highly characteristic and nonrandom [43]. Generally, tumor suppressor genes often have loss of functional mutations or a loss of heterozygosity or reduced gene expression [44]. Regarding *FOXL2*, previous papers have shown differential regulation between its wildtype and mutants [45, 46]. Rosario et al. review the related papers and suggest that mutant *FOXL2* maintains some of the transcriptional activity of the wildtype allele, but there is a subtle alteration of the expression in a unique suite of cancer-related genes, and they suggest that it is an oncogene or tumor suppressor, depending on the context related to the GCTs subtype [44].

Cervical cancer is the second most commonly diagnosed cancer and the third leading cause of cancer death among females in less developed countries, and there are an estimated 311,365 deaths from cervical cancer worldwide in 2018 [47]. The main risk factor for this cancer is persistent infection with high-risk human papillomavirus (HPV-HR) types [48]. In the clinic, concurrent administration of carboplatin and paclitaxel combined with surgery and radiotherapy is the standard treatment for cervical cancer patients with high risk factors [49]. However, this therapeutic method is often defeated by cisplatin resistance and peritoneal metastasis, while our results may suggest a new approach.

## Conclusion

**Fig. 5:**
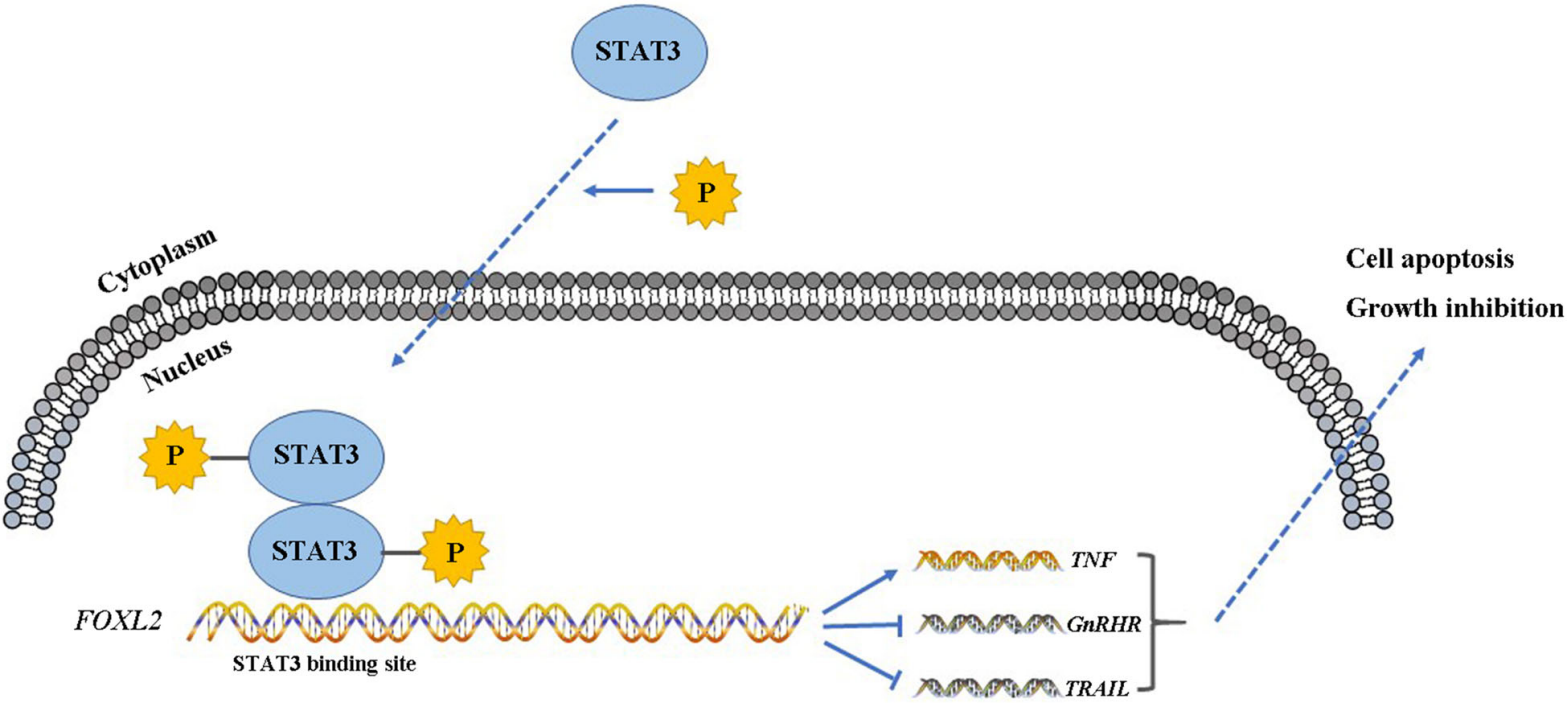
Summary of STAT3-FOXL2 signaling function in HeLa cell growth and apoptosis. Phosphorylated-STAT3 enters the cell nucleus from the cytoplasm and binds to the confirmed new binding sites, which are located in the promoter region of FOXL2, then up-regulates the expression of *TNF*, and down-regulates the expression of *GnRHR* and *TRAIL*. Finally, the cancer cell growth inhibition and apoptosis are inhibited

Our results in this paper demonstrate that *FOXL2* is regulated by STAT3, thereby providing a new STAT3-FOXL2 pathway. Meanwhile, the new pathway has important functions in HeLa cell growth and apoptosis, indicating the new role of FOXL2 in cervical cancer growth (Fig. 5), and these results may provide new insight into the crucial transcription factor FOXL2.

## Methods

### Materials

Fetal bovine serum (FBS) was obtained from HyClone (Logan, UT). Dulbecco’s Modified Eagle’s Medium (DMEM) was purchased from HyClone (GE Healthcare Life Sciences, Logan, UT, USA). Sequence-specific Silencer Select siRNAs targeting human STAT3 mRNA (Catalogue #4390824/s743, sense: 5′-GCCUCAAGAUUGACCUAGATT − 3′, antisense: 5′-UCUAGGUCAAUCUUGAGGCCT-3′) or FOXL2 mRNA (Catalogue #4392420/s2068, sense: 5′-CGAAGUUCCCGUUCUACGATT-3′, antisense: 5′-UCGUAGAACGGGAACUUCGCG-3′) were purchased from Thermo Fisher Scientific (Waltham, USA). The scrambled Silencer Select Negative Control #1 siRNA (Catalogue #4390843), Silencer Select GAPDH Positive Control (Catalogue #4390849) and BLOCK-IT Alexa Fluro Red Fluorescent Control (Catalogue #14750100), both purchased from Thermo Fisher Scientific (Waltham, USA). Anti-STAT3 (phospho Y705) monoclonal antibody (Catalogue #ab76315), anti-FOXL2 monoclonal antibody (Catalogue #ab188584) and anti-GAPDH monoclonal antibody (Catalogue #ab181602) were all purchased from Abcam (Cambridge, MA, USA). *Pst* I (Catalogue #R0140T) and *Xma* I (Catalogue #R0180S) were purchased from NEB (USA), The inhibitor, WP1066, was purchased from Merck Company (Merck KGaA, Darmstadt, Germany), and dissolved in dimethyl sulfoxide (DMSO; Solarbio, Beijing, China) as a stock solution.

### Cell line and culture

The HeLa cell line, which was obtained from our previous research materials and stored in the Shandong Key Laboratory of Medical and Health Sciences (Shandong, China), was cultured in high-glucose Dulbecco’s modified Eagle’s medium (DMEM; Hyclone; GE Healthcare Life Sciences, Logan, UT, USA) supplemented with 10% fetal bovine serum (HyClone; GE Healthcare Life Sciences) at 37 °C in a humidified environment with 5% CO2. Before analysis, HeLa cells were incubated with human IL-6 to stimulate the STAT3.

### Transient transfection

For transient transfection using Invitrogen Lipofectamine 3000 (Thermo Fisher Scientific, Inc., Waltham, MA, USA), the Lipofectamine 3000 Reagent was diluted in Opti-MEM medium (Thermo Fisher Scientific, Inc.), and the transfection complexes were prepared by diluting the siRNA in Opti-MEM medium. Subsequently, diluted siRNA was added to each tube of diluted Lipofectamine 3000 Reagent (1:1 ratio) and incubated for 5 min at room temperature. Different siRNA-lipid complexes (0.25 μg per well) were then added to the HeLa cells in 12 well plates (70–90% confluent, 37 °C, 5% CO2) separately, and cells were used for the subsequent experiment after transfection for 24 h. HeLa cells that were transfected with negative siRNA (Thermo Fisher), and non-siRNA were used as controls, and the BLOCK-IT Alexa Fluro Red Fluorescent Control (Thermo Fisher) with doses ranging from 0 to 50 nM was used in the pre-transfection for detecting the optimum efficiency.

### RNA extraction and cDNA synthesis

Total RNA was extracted from the cells with the TRIzol reagent (TaKaRa, Japan) according to the manufacturers’ protocol and then treated with DNase I (RNase-free, Promega). Total RNA was subjected to first-strand cDNA synthesis with a RevertAid First Strand cDNA Synthesis Kit (Fermentas, USA) according to the manufacturer’s protocol.

### Gene expression analysis by qRT-PCR

PCR was carried out in a 20 μl reaction containing 1 × SYBR Green PCR Master Mix (TAKARA, Japan), 400 nM primers (for each forward and reverse primer), and 1 μl of the reverse transcription reaction. Quantitative analysis was performed using the Eppendorf Realplex system with PCR conditions of 94 °C for 15 s, 61 °C for 30 s and 68 °C for 35 s for 40 cycles. The absence of primer-dimer formation was examined in single and no-primer controls. Each sample was examined in triplicate using relative quantification analysis. This method normalizes the expression of the specific gene versus the control reference with the formula 2-ΔΔCT, where ΔCT = CT specific gene - CT reference gene, and ΔΔCT = ΔCT - arbitrary constant. The threshold cycle value is defined as the PCR cycle number that crosses an arbitrarily placed threshold line. All of the primers used in qRT-PCR are shown in Table 1 [10].

**Table 1:** Primers used in qRT-PCR

Primer name	Sequences (5′-3′)
hTRAIL-F	GCGCAGCGAGTGGGACAGAG
hTRAIL-R	GGCACTGGGTCCGTGCTGTC
hTNF-F	CCAAATGGGGGAGTGAGAGG
hTNF-R	AAAGGCAAAGACCAAAGAAAATGA
hFas-F	TGAAGGACATGGCTTAGAAGTG
hFas-R	GGTGCAAGGGTCACAGTGTT
hGnRHR-F	ACCGCTCCCTGGCTATCAC
hGnRHR-R	GACTGTCCGACTTTGCTGTTGCT
hGAPDH-F	GAGTCAACGGATTTGGTCGT
hGAPDH-R	GACAAGCTTCCCGTTCTCAG

### Western blotting

Total cellular lysate preparation for Western Blot analysis was performed as described previously [21]. Generally, proteins extracted from cells were boiled in 1 × SDS PAGE loading buffer (TAKARA, Japan), and then separated on 10% SDS-PAGE gels. Then the protein was blotted onto PVDF membrane (Millipore, USA) using electrophoretic transfer. Immunoblotting was performed with antibodies against p-STAT3 (1:2000, Abcam, USA), FOXL2 (1:8000, Abcam, USA), GAPDH (1:8000, Abcam, USA), and ALP-conjugated goat anti-rabbit (ZSGB-BIO, Beijing, China). An enhanced NBT-BCIP chromogenic substrate kit (TIANGEN, China) was used for immunodetection.

### ChIP assay

We used a Pierce TM Agarose ChIP Kit (Thermo Fisher Scientific, Waltham, USA) in accordance with the manufacturer’s instructions. Briefly, cells were first treated with IL-6 for cross-linking with 1% formaldehyde for 10 min at room temperature, then 1 × glycine solution was added, and the cells were incubated at room temperature for 5 min. Then, the cells were harvested and incubated on ice for 10 min in lysis buffer. Nuclei were pelleted and digested by restriction enzymes (*Pst* I and *Xma* I) at 37 °C for 15 min. Following sonication and centrifugation, sheared chromatin was incubated with anti-STAT3 antibody (Santa Cruz) overnight at 4 °C. Then, protein-A/G beads were added and the chromatin was incubated for 2 h in rotation. Antibody-bound protein-DNA complexes were eluted and subjected to PCR analysis. The primer sets used to amplify the human FOXL2 promoter were primer A: 5′ AAGGCACTGTTAAGGAGCAC-3′, primer B: 5′- ATTTGAACAAGTCCCTCCCTC-3′, and primer C: 5′- CTCAGAAACACAGGTATGCAC-3′, which generated a 426-bp product using primers A and C, and a 255-bp product using primers B and C.

### EMSA

The electrophoretic mobility shift assay (EMSA) was produced according to the manufacturer’s protocol (Catalogue #20148, Thermo Fisher Scientific, Waltham, USA). Briefly, nuclear extracts were prepared using NE-PER Nuclear and Cytoplasmic Extraction Reagents (Catalogue #78833, Thermo Fisher Scientific, Waltham, USA), and incubated with biotin-labeled FOXL2 promoter DNA probe (5′ Biotin-GCCTGATGTT**TGTCTTCCCAGTCTGT**GGCAA-3′) in binding buffer for 30 min on ice. Following incubation, the samples were separated on a 5% polyacrylamide gel in Tris-borate EDTA, transferred onto a nylon membrane, and fixed on the membrane by UV-cross-linking. Finally, the membrane was exposed to X-ray film for 2–5 min. The 25-fold and 100-fold excess cold probes combined with biotin-labeled probes were used as competition controls. To confirm the results of protein-DNA binding, 1 μg of rabbit anti-human phosphotyrosine STAT3 antibody was incubated with the nuclear extracts for 30 min on ice before adding the biotin-labeled DNA probe.

### Flow cytometry detection of cell apoptosis

For flow cytometry (FCM) detection, an Annexin V-FITC/PI Apoptosis Detection Kit (TransGen Biotech, Beijing, China) was used to detect the early apoptosis levels. The experiment was performed according to the manufacturer’s protocol. The negative control (without Annexin V and PI) and positive controls (with Annexin V or PI) were also designed according to the protocol. After the examples were prepared, the fluorescence signal was detected within 1 h by FCM (BD). Annexin V-FITC was detected at a 488-nm excitation wavelength and a 530-emission wavelength. Propidium iodide was detected at 488 nm excitation wavelength and a 630-nm emission wavelength.

### Real-time cellular viability assay by RTCA

The method of cell viability assay using RTCA was carefully described as in our previous paper [26].

### Statistical analysis

Statistical analysis was performed using the Data Processing System 7.05 software (DPS 7.05, http://www.dpsw.cn/, Zhejiang University, China). Statistical significance was tested using Duncan’s test at 0.05, 0.01 or 0.001 probability levels.

## Additional file

Additional file 1: The prediction of *cis*-elements in the promoter region of FOXL2. (DOCX 18 kb)

## Data Availability

The datasets used and/or analyzed during the current study are available from the corresponding author on reasonable request.

## Abbreviations

BPES: Blepharophimosis-ptosis-epicanthus inversus syndrome
ChIP: Chromatin immunoprecipitation
EMSA: Electrophoretic mobility shift assay
FCM: Flow cytometry
FOXL2: Forkhead box L2
GCTs: Granulosa cell tumors
PCOS: Polycystic ovary syndrome
PI: Propidium iodide
PS: Phosphatidylserine
RTCA: Real-time cellular assay
STAT3: Signal transducer and activator of transcription 3
