# Supplementary material for: New STAT3-FOXL2 pathway and its function in cancer cells
Source: BMC Mol Cell Biol. 2019 Jun 20;20:17. doi: 10.1186/s12860-019-0206-3 (PMC6587274; doi:10.1186/s12860-019-0206-3)
Supplement: Supplementary file 1 — The prediction of cis-elements in the promoter region of FOXL2. (DOCX 18 kb) [file 12860_2019_206_MOESM1_ESM.docx]

-2031 CAGGGATTCT GCGGAAGTGC TGCTTCCCCC ATGTCTGTTG GGTGCAGTGG GAGACCGCCC

-1971 ATAAAAAAAA CCAAGCTACT TTGGGGATAG CCTTGCTCCC TCTCATCCTT GTGACCAGGC

-1911 AAGGGCTGCC CTGCTGACCT CTCTGGTGTC ATCTTCAAGA TGCTGAATTC CAAAGAGCCC

-1851 TACCACCCTT GTCCTCCCTC CCTTTCATCT CAGAA**TGCAG ATCA**GAGGCC TGAGTGAGCA

**GATA**

-1791 GCAGACAACA CTTGTAGATG GCCTGTGGAT GCTGGAACCA CAGGCCGCTG AAGGCTCCTT

-1731 GGGCTTCAGG AGAACCCTCA GAGTCTAAAC AGCCTGGGAG GAAGAGGCAG TGACAGGCA**T**

-1671 **TGAGTCAG**CC ACTCCAGACA TGCCAATCAC TAATGCACAG CTATATGACA TCCATTCAGT

**AP1**

-1611 CCACCTTCCT CCATAAGGTG GCCTTGC**CCC CCTCCTC**AGC ACCAAGGAGT TTGGGGCTTG

**SP1,SP3**

-1551 AGGATTCAAA ACACTGGCCA TCTCTCTCAT ACTTTATGAC TGCAGACTTT TTGTTTAGGC

-1491 CAAGTTAAAA GGCACTGTTA AGGAGCACAC TGTTAAAAGG CACACCTGAT TTTCCCTGAA

-1431 ACAATCTCTG TACATGCTGA AATAATTGGA TTCCCATCAG AATTCTGCTT AAGGTAGGTG

-1371 ACAACTAACT CCTGATTCTT TGGAGAAAGA TCAGAACAAA TGTCTCATCA GGAGAGAGAT

-1311 TTGAACAAGT CCCTCCCTCA GCCTGATGTT **TGTCTTCCCA GTCTGT**GGCA AAATACATAC

**STAT3**

-1251 ACTGGGAGGG AAAGAGGCAT CGAAGAATTG TGAAAGAAAG GAAGAAAGGG GGGAAGGAAA

-1191 GAAGTAAGAA AGGAGAAAAA GAGAAGATGC TATATCCACC AGCTACATTT ACTCTAATAC

-1131 TTGATGAACA TATTACATAA CTATCAGTAT AAGTGTGTTT TACCTCGTAA ATACAAATCT

-1071 CACACCTTGT ACATGTGCAT ACCTGTGTTT CTGAGCAGCA CACATGCACC TGGTATCTCA

-1011 CCCATCTCAA GGACAGCTCC TCTCCATGAC TTCCATATGT GACCCCTGTG CACACACATA

-951 CACAGTCCCG CAAAGTCCCG GGCAACAGCC CCGCCTGTGT TAGTGCAATA GCTGAGGGGC

-891 ATCTACTTGG TGCTGTTAAA GCACACATCC AACCTTCACT CCAACCCCGT AGGATTAGTA

-831 CACGGATTCC TGGTTCCTAG ATCCACTGGT AACGCAGGGA GAGGCAGAGG **TGATTCAGG**C

**TPA**

-771 GGCGCCCCTG ACGACTATTT CCAGAGATCC TCTCCCCTGC CCCATTCCAG CCCAGTCACT

-711 CTGAGAGCGC GAGGGACAGG CCAGGCCGAG GCTTCTTCGT ACAGTCACAC CCGTTTCTGG

-651 GCTGACTTTC CCAAAGCCTC AGAGCAGCTC TCCCCACCCT ACCCCGTCCA GCCCCCTCGC

-591 CGTCTCGCCC CTCCCCAGCC CCGAGCTAGA AGTTCAGCCT CAAGACGGCA GCAAACGGCA

-531 GAGCAAAGGA GTCGTTTTCT TTCACCTGAA AGCCGCGAGG AGGCTTGGAG CGCCTTTCCT

-471 CGCTGGGGCC CGAGCTTCCT GGGCTTTGGC CGGGTGCGGG CAGACCCCAC CGGGGTCCTC

-411 GTCATCTCCC AGCCCAGCCC GCAGAGCGAG TACCGGCAGA TTTCAAGGGC GCGTGAGCCT

-351 GGCTGTCGGC TGGGCCCCTG AGGCTC**GCTG GGCGGGGGCA G**GCCGGTCCA GGCTGTGCGG

**ETF**

-291 GGCGTTTACA AAAAGTGACT TGGAGATGAA CTCGCCCGTG CGCGGCTGGC CGCCCCGCTA

-231 TAGGGGCGAA GGCGCCTGAC GCAAGCGGAA CTCGGTGGAG CCCATACGAA TCAGAACAGA

-171 GCGAGGCTCC TGGCGCACTA GGGACTCCAG GAGGCAGCTC CGCCAGAGAC GCGGGTCGTG

-111 CTTCGGGAAA CCGGGGG**GCG GGGGGAGGGG A**AGAGCGCAG AAAAGAAAAC CCACCAAGGC

**AP1/SP2**

-51 GGGGACTGGC CTGAGCGGGG AGGGGCGGCG AGGCCGGAGC CCCTCTCTGT T**G**GGCGGACT

**+1**

+10 CCCCATGGCC AGAGGCTGAG CTCCACTCCC GCCGGCCGCT CCCTAGGGGA AGGGGAAGGA

+70 GAGGGGAGAG CAGCGACAGG CCTCCAGCAA GCAAGCGCGG GCGGCATCCG CAGTCTCCAG

+130 AAGTTTGAGA CTTGGCCGTA AGCGGACTCG TGCGCCCCAA CTCTTTGCCG CGCCAGCGCC

+190 TGGAGCGGAG AGCAGAGGCG GCCCGGCCGC GGCGCGCCGG CTTTGTC**ATG** ATGGCCAGCT

+250 ACCCCGAGCC CGAGGACGCG GCGGGGGCCC TGCTGGCCCC AGAGACCGGT CGCACAGTCA

+310 AGGAGCCAGA AGGGCCGCCG CCGAGCCCA

| ***Cis*-element** | **Position** | **Sequence (5’-3’)** |
| --- | --- | --- |
| *AP1* | －360 (+) | TTGAGTCAG |
| *AP2/Sp1* | －1951 (－)  －1935 (+) | TCCCCTCCCCCCGC/  GGGGCGGGGG |
| *ETF* | －1721 (－) | CTGCCCCCGCCCAGC |
| *Erythrocyte-specific protein* | －1933 (+), －1934 (+),  －1935 (+) | GGGGGGCGGGGGGAGG/GAGGG/AGGGG |
| *GATA* | －261 (+) | TGCAGATCA |
| *GBF* | －1941 (+) | GGGGGAGGGG |
| *TPA* | －1251 (+) | TGATTCAGG |
| *SP1/SP3* | －457 (－) | GAGGAGGGGG |
| *STAT3* | －767 (－) | CACAGACTGGGAAGACA |
